# Effect of virgin olive oil versus piroxicam phonophoresis on exercise-induced anterior knee pain

**Published:** 2016

**Authors:** Babak Nakhostin-Roohi, Faegheh Khoshkhahesh, Shahab Bohlooli

**Affiliations:** 1 *Department of Exercise Physiology, Islamic Azad University-Ardabil Branch, Ardabil, Iran*; 2 *Department of Exercise Physiology, University of Mohaghegh- Ardabili, Ardabil, Iran*; 3 *Department of Pharmacology, School of Pharmacy, Ardabil University of Medical Sciences, Ardabil, Iran*

**Keywords:** *Phonophoresis*, *Olive oil*, *Piroxicam*, *Anterior Knee Pain*, *Topical application*

## Abstract

**Objective:**

The main purpose of this study was to evaluate the effects of virgin olive oil phonophoresis on female athletes' anterior knee pain (AKP).

**Materials and Methods:**

A double blinded randomized clinical trial was conducted. Ninety-three female athletes suffering from AKP voluntarily participated in this study. Patients were randomly assigned into olive oil (n=31), piroxicam (n=31) or base gel phonophoresis (n=31) groups. At the baseline visit, the Western Ontario and McMaster Universities Osteoarthritis Index (WOMAC) questionnaire was filled by subjects who were then treated with olive oil, piroxicam or pure phonophoresis for 12 sessions. After 6 and 12 sessions of physiotherapy, subjects filled the questionnaire again. Main outcomes were significant improvement in pain, stiffness, physical function, and total WOMAC scores.

**Results:**

Although, there was a significant reduction in symptoms of AKP at the end of the therapy in all groups (p<0.05), but in olive oil group, this improvement was seen after 6 sessions of treatment (p<0.001). A significant difference between olive oil group and piroxicam and/or phonophoresis group was observed after 6 sessions of therapy (p<0.05).

**Conclusion:**

It could be proposed that phonophoresis with virgin olive oil is as effective as piroxicam gel on lowering WOMAC scores of AKP in female athletes and also has several beneficial properties including faster effect and shorter duration of therapy. The exact mechanism of beneficial action of virgin olive oil on AKP is not clear and requires further studies.

## Introduction

Anterior knee pain (AKP) is a characteristic symptom in which pain is usually experienced in front of the knee and worsens by sitting for long periods or stairs climbing and descending (Nimon et al., 1998 ▶). AKP is a common complaint among athletes and active young individuals (Davis and Fulkerson, 1999 ▶). Injuries of the anterior knee can be caused by two mechanisms: acute traumatic and overuse injuries. Most of the knee-structures can be injured by both mechanisms (Llopis and Padrón, 2007 ▶). Trauma may be acute but is more frequently the result of repetitive overuse. Training errors (usually too much exercise too soon), mal-alignment of the hip or the extensor mechanism including the patella and the feet, deficiencies in strength and flexibility, ill-fitting shoes and uneven training surfaces are the most common problems that contribute to AKP (Taunton and Wilkinson, 2001 ▶). Although, a major focus of treatment is correction of biomechanical factors that led to AKP (Adams, 2004 ▶), the use of therapeutic modalities such as cryotherapy, thermotherapy and ultrasound during the rehabilitative process is an important component in the effort to return the athlete to full function. Modalities can be used to reduce pain and edema, increase mobility, and effectively deliver medications across the skin (Stracciolini et al., 2007 ▶).

Phonophoresis uses ultrasound to drive a drug through the skin and into underlying tissues (Kleinkort and Wood, 1975 ▶; Michlovitz, 1990 ▶). In theory, ultrasound can enhance the transdermal delivery of certain pharmacologic agents to skeletal muscle tissue, bursa, tendons, and so on. Thus, phonophoresis offers the potential advantage of delivering a pharmacologic agent in a relatively safe, painless, and easy manner to structures that lie somewhat deep within the body (Ciccone et al., 1991 ▶). Phonophoresis has been used to administer various drugs, including local anesthetics and antibiotics (Byl, 1995 ▶). This technique has also been used successfully to deliver anti-inflammatory medications to inflamed subcutaneous tissues (Byl, 1995 ▶; Griffin and Touchstone, 1968 ▶; Oziomek et al., 1991 ▶).

Recently, the beneficial properties of virgin olive oil such as increasing serum HDL (Martinez-Beamonte et al., 2013 ▶), anti-aging (Menendez et al., 2013 ▶; Vazquez-Martin et al., 2012 ▶), anti-inflammatory, immunomodulatory, anti-proliferative and anti-apoptotic effects (Sanchez-Fidalgo et al., 2013 ▶) have been documented. It was suggested that these effects may be due to the antioxidant properties attributed to its components, including the monounsaturated fatty acid and oleic acid (Owen et al., 2000 ▶; Stark and Madar, 2002 ▶). Although the composition of olive oil is complex, the major groups of compounds thought to contribute to its health benefits include oleic acid, phenolics, and squalene (Owen et al., 2000 ▶), all of which have been found to inhibit oxidative stress (Garrido et al., 2013 ▶; Waterman and Lockwood, 2007 ▶). Researchers observed the beneficial effects of olive oil on rheumatoid arthritis following oral consumption (Kremer et al., 1990 ▶) or topical application (Bohlooli et al., 2012 ▶). In 2005, (-)-oleocanthal, the dialdehydic form of (-)-deacetoxy-ligstrosideaglycone present in freshly-pressed extra virgin olive oil was discovered to have the properties of a non-steroidal anti-inflammatory drug (NSAID) (Beauchamp et al., 2005 ▶) which may be contributed to its anti-rheumatoid and pain reliving action.

Although physical therapy modalities such as phonophoresis are used by physiotherapist in outpatient clinics, scientific evidence to support their use is insufficient and sometimes controversial (Bolin and Md, 2003 ▶). Accordingly, we conducted a prospective double blinded clinical trial of short term phonophoresis of virgin olive oil versus piroxicam gel and/or base gel ultrasound in management of exercise-induced AKP in female athletes.

## Materials and Methods

This study was conducted at Shafa physiotherapy clinic, Ardabil, Iran. The study was approved by the Ethics Committee of Islamic Azad University-Ardabil Branch, and performed in accordance with the ethical standards of the declaration of Helsinki.

**Patients**

In this study, 93 female athletes (age: 26.13 ± 5.87 yr; weight: 58.67 ± 6.35 kg and height: 162.62 ± 5.76 cm) participated. Only female subjects were selected in order to eliminate any potential gender-related differences. Subjects had been symptomatic for at least 6 months with the knee as the primary site of pain or disability which had not responded adequately to treatment with non-steroidal anti-inflammatory drugs. All patients had a minimum score of 29 on the Western Ontario and McMaster Universities Osteoarthritis Index (WOMAC). Patients were excluded from the study if they had any systemic illness or abnormal laboratory test results, dermatological problems, skin allergy to NSAIDs, local ischemic problems, atrophic or scarred skin, bleeding dyscrasias or had been on any physiotherapy program or received intra-articular injections in the preceding year, or had symptoms and signs of acute synovitis. Patients were assessed by expert orthopedist or physiotherapist by history and a detailed physical examination. All patients were initially questioned about age, weight, height and duration of knee pain. In patients in whom both knees were symptomatic, both knees were chosen as the target knee. Also, patients did not receive any treatments at least for the last 2 months. 

Subjects were randomly assigned into olive oil, piroxicam or base gel groups. Phonophoresis was accomplished in all groups. The WOMAC questionnaire was used to measure pain, stiffness and physical function. Questionnaires were filled by all patients before and 6 and 12 sessions after treatment. The WOMAC index is technically developed for osteoarthritis of knee, but based on the type of questions, it could be of value in evaluating any other knee movement problems. Therefore, we found it useful for assessing the effect of interventions on AKP. 

**Intervention**

The physiotherapy program was conducted six times a week for two weeks, excluding weekend, for a total of 12 sessions. During the therapy sessions (while the patients were lying supine), hot packs wrapped in toweling were placed on the target knee for 20 min, followed by deep heating with ultrasound application. In the piroxicam group, piroxicam gel (0.5%, Razak Inc., Iran) was applied from the blinded 30-gram tube over the target knee. In contrast, in the olive oil group, virgin olive oil (olives were obtained from gardens in Gilan province, Iran and packaged as oil in 30-gram tubes) was applied from the blinded 30-gram tube over the target knee. In each visit, 10 grams of olive oil or piroxicam gel and/or base gel was applied to target knee. The tubes were coded by randomly-generated numbers and were identical in appearance. Patients and visiting clinician were unaware of the content of tubes. Each patient was sequentially allocated to the next assigned container according to the randomization code. No patient was taken out of turn. The final assessor was blind to groups and interventions. Randomization data were kept strictly confidential by another person. Continuous ultrasonic waves with 1 MHz frequency and 1 Watt/cm^2^ power were applied with a 4 cm diameter applicator (Phy-action, Netherlands) to the super-medial and lateral parts of the knee by the same therapist stroking the applicator in circular movements. The transducer head was applied to the therapy region at right angles to ensure maximum absorption of the ultrasound energy. Ultrasound therapy lasted 5 min for each knee in each session. 

**Statistical analysis**

All demographic and quantitative data were expressed as mean ± standard error and p<0.05 was considered statistically significant. An independent two-way analysis of variance (ANOVA) with repeated measures was used to compare results among groups and over time.

## Results

**Patient characteristics**

The subjects were randomly assigned into treatment with virgin olive oil, piroxicam or base gel phonophoresis. Baseline characteristics of these 93 patients are given in Table 1. There were no significant differences with respect to age, weight, and height among groups (p< 0.05). 

**Table1:** Baseline demographic characteristics of patients with AKP by study group

	**Base gel** **N=31**	**Piroxicam** **N=31**	**Olive oil** ** N=31**
**Age (yr)**	27.15 (1.67)	26.23 (1.83)	26.00 (1.57)
**Weight (kg)**	61.00 (2.30)	58.15 (1.97)	59.27 (1.68)
**Height (cm)**	1.62 (1.62)	164.23 (1.28)	160.73 (1.99)

Values are mean ± SE

**Clinical outcomes**

**Figure 1:**
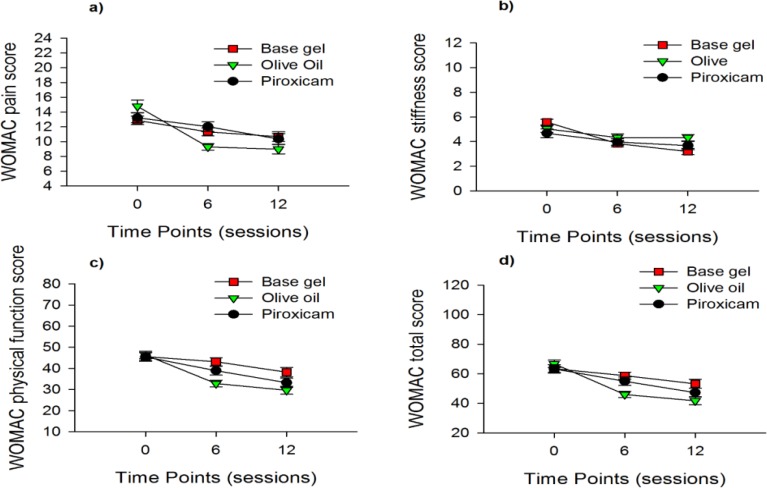
WOMAC a) pain; b) stiffness; c) physical function and d) total scores over sessions in patients with AKP treated with base gel ultrasound, virgin olive oil or piroxicam phonophoresis. WOMAC: The Western Ontario and McMaster Universities Arthritis Index; AKP: anterior knee pain

The primary outcome of our study was significant improvement in pain, stiffness, physical function, and total WOMAC scores at the end of the therapy program as compared to baseline scores (Figure 1). According to the results of questionnaire, phonophoresis with virgin olive oil was able to significantly reduce pain, physical function and total scores even after 6 sessions (p<0.001) showing faster effect of olive oil compared to piroxicam or base gel phonophoresis. At the end of the 6th session, only olive oil group showed significant decrease in all of the WOMAC sub-scores as compare to baseline (p<0.001, Figure 1). Patients in piroxicam group showed a significant decrease only in WOMAC physical function score at the end of the 6th session (p< 0.05, Figure 1d). Patients in olive oil group showed a significant decrease in WOMAC pain (p<0.01) and total (p<0.05) scores versus piroxicam group and physical function (p<0.001) and total (p<0.01) scores as compared to base gel phonophoresis after 6 sessions of therapy. At the end of the 12th session, WOMAC pain, physical function and total scores were significantly lower than base line in all three groups (p<0.05, Figures 1a, c and d). Group with olive oil phonophoresis showed significant decrease in WOMAC physical function, stiffness and total scores as compared to base gel phonophoresis at the end of the 12th session (p<0.05). Regarding WOMAC pain sub-score, there was no significant difference among groups after 12 sessions of treatment.

## Discussion

In our previous work, superiority of topical virgin olive oil over piroxicam gel was shown in osteoarthritis of the knee (Bohlooli et al., 2012 ▶). The present study was designed to determine whether phonophoresis with virgin olive oil could have an influence on AKP in female athletes. The primary novel finding is that the phonophoresis with olive oil reduces the pain and stiffness, improves physical function and offers shorter duration of treatment than base gel or piroxicam. Although, phonophoresis in all groups was able to significantly decline symptoms of AKP after 12 sessions, but in olive oil group, WOMAC pain, stiffness, physical function and total scores significantly declined after 6 sessions showing faster effect of phonophoresis with olive oil. Several *in vitro* and *in vivo* studies have examined the anti-inflammatory properties of olive oil (Perona et al., 2006 ▶). Recently, we also found that phonophoresis with virgin olive oil is beneficial in low back pain control in female athletes (Nakhostin- Roohi and Bohlooli, 2014 ▶) and it is more efficient than phonophoresis with base ultrasound gel. Also, other researchers showed that application of extra virgin oil with mild physical activity can prevent cartilage degeneration in an osteoarthritis model (Musumeci et al., 2013 ▶). 

It is believed that olive oil exerts its biological benefits mainly via its anti-oxidants constituents (Waterman and Lockwood, 2007 ▶). The composition of olive oil is complex (Owen et al., 2000 ▶) and recently a secoiridoid derivative, oleocanthal – the dialdehydic form of deacetoxy-ligstrosideaglycone– was identified with an activity similar to that of NSAIDs (Beauchamp et al., 2005 ▶). This compound which has an extreme irritating effect on the throat, demonstrated an anti-inflammatory activity and an inhibitory effect on cyclooxygenase enzymes (Beauchamp et al., 2005 ▶) which may be responsible for some of the effects of topical application of virgin olive oil on AKP. It is likely that the presence of several types of anti-oxidants and anti-inflammatory agents in virgin olive oil is responsible for faster effect of olive oil as compared to piroxicam phonophoresis. On the other hand, the heating effect of ultrasound which encourages regional blood flow and increases connective tissue extensibility should not be ignored. Non-thermal effects are less understood and include molecular vibration, which increases cell membrane permeability and thereby enhances metabolic product transport (DeLisa, 1988 ▶). Nevertheless, the exact mechanism of the virgin olive oil remains to be addressed and requires further studies.

It could be proposed that phonophoresis with virgin olive oil is superior or at least as effective as piroxicam gel on lowering WOMAC scores of AKP in female athletes. Also, virgin olive oil offers shorter duration of therapy and faster and better response.

## Note

*Please cite this paper as: *

Nakhostin-Roohi B, Khoshkhahesh F, Bohlooli S. Effect of virgin olive oil versus piroxicam phonophoresis on exercise-induced anterior knee pain. Avicenna J Phytomed, 2016; 6 (5): 535-541.
